# Stabilization of *Cereibacter sphaeroides* Photosynthetic Reaction Center by the Introduction of Disulfide Bonds

**DOI:** 10.3390/membranes13020154

**Published:** 2023-01-25

**Authors:** Georgii Selikhanov, Anastasia Atamas, Diana Yukhimchuk, Tatiana Fufina, Lyudmila Vasilieva, Azat Gabdulkhakov

**Affiliations:** 1 Institute of Protein Research, Russian Academy of Sciences, Institutskaya 4, 142290 Pushchino, Moscow Region, Russia; 2 Federal Research Center Pushchino Scientific Center for Biological Research PSCBR, Institute of Basic Biological Problems, Russian Academy of Sciences, Institutskaya 2, 142290 Pushchino, Moscow Region, Russia

**Keywords:** protein stabilization, disulfide bonds, photosynthetic reaction center, integral membrane protein, site-directed mutagenesis, crystal structure

## Abstract

The photosynthetic reaction center of the purple nonsulfur bacterium *Cereibacter sphaeroides* is a useful model for the study of mechanisms of photoinduced electron transfer and a promising component for photo-bio-electrocatalytic systems. The basic research and technological applications of this membrane pigment-protein complex require effective approaches to increase its structural stability. In this work, a rational design approach to genetically modify the reaction centers by introducing disulfide bonds is used. This resulted in significantly increasing the thermal stability of some of the mutant pigment-protein complexes. The formation of the S-S bonds was confirmed by X-ray crystallography as well as SDS-PAGE, and the optical properties of the reaction centers were studied. The genetically modified reaction centers presented here preserved their ability for photochemical charge separation and could be of interest for basic science and biotechnology.

## 1. Introduction

The photosynthetic reaction center (RC) of the purple bacterium *Cereibacter sphaeroides* (recently renamed from *Rhodobacter sphaeroides*) is a photosensitive membrane pigment-protein complex that serves as a convenient and informative test model for studying the mechanisms of electron transfer, photosynthesis, and pigment-protein interactions. It is a well-studied, relatively stable integral membrane protein (IMP) with established protocols for its expression, isolation from membranes, and purification. The RC from *С. sphaeroides* consists of three protein subunits and ten cofactors arranged in two membrane-spanning branches, A and B. The RC contains two bacteriochlorophylls (BChl), P_A_ and P_B_, combined into a special pair P, two monomeric BChls, B_A_ and B_B_, two bacteriopheophytins (BPhe), H_A_ and H_B_, two quinones, a non-heme Fe atom, and a spheroidene molecule [1]. This bacterial reaction center shares considerable similarities with the photosystem II of plants, algae, and cyanobacteria [2] and has therefore served for many years as a structural and functional model for the study of the more complex photosystem II.

One of the most common methods to study the mechanisms of photochemical processes in the reaction centers is via amino acid substitutions. This method allows new data to be obtained, but such substitutions can often lead to decreased structural stability of the protein [3]. Studies on mutant forms of the reaction center with reduced stability prove difficult due to the higher denaturation rate of the purified complexes, especially when X-ray protein crystallography is to be used, where the stability of the macromolecule under study is one of the main factors for successful crystallization [4]. Increasing the stability of complexes by introducing compensatory mutations that do not affect their function is one of the possible methods to study such objects.

Another aspect where the overall stability of the reaction center is of great importance is the potential technological applications in the field of photo-bioelectrocatalysis. Both isolated reaction centers and intact cells of purple bacteria are promising catalysts that convert light energy into chemical energy in charge-separated states. Primary charge separation and subsequent electron transfer across photosynthetic membranes occur with a quantum yield approaching 100% [5]. However, to make photo-bioelectrochemical systems appealing to industry, it is critical to improve the stability of their components to enable long-term application and to develop cost-effective systems (both in terms of their assembly and operation) [6].

In this regard, it is important to have effective approaches to improving the structural stability of reaction centers to facilitate their basic studies and industrial applications. Recently, the effect of various detergents and osmolytes on the thermal stability of bacterial RC complexes has been studied [7]. Sodium cholate has been shown to have a significant stabilizing effect on the structure of native and genetically modified RCs. However, it is not always possible to change the storage conditions of the reaction centers due to the specifics of the experiment or biotechnological process in which the complex is used.

H-bond networks have been shown to play an important role in the stability of reaction centers [8]. For *C. sphaeroides* RC, the H-bond of the acetyl group of each of the bacteriochlorophylls in the dimer ensures the stability of the complex at elevated temperature and pressure [9,10]. The introduction of hydrogen bonds to stabilize the RC is possible but has two major drawbacks: (1) The complexity of hydrogen bond interactions in membrane complexes makes it difficult to design appropriate substitutions and predict their influence on the RC structure; (2) There are hydrogen bonding networks located near the electron transport cofactors of the RC, and their manipulations may affect the functional activity of the complex.

Here we present an approach for stabilization of the photosynthetic reaction center of *Cereibacter sphaeroides*, which is the formation of disulfide bonds between its α-helices. Investigations were undertaken into the possibility of S-S bond formation at different sites and also the effects of their introduction on the thermal stability and functional activity of the complex.

## 2. Materials and Methods

### 2.1. Mutagenesis

A genetic system for site-directed mutagenesis consisting of the *C. sphaeroides* DD13 strain [11] deficient in RC and antennae systems synthesis and the pRK plasmids described elsewhere [12] was used. Mutations were introduced into *puf*-operone using PCR oligonucleotides via the QuikChange plasmid mutagenesis protocol as described in [13]. The nucleotide changes were confirmed by DNA sequencing. The altered pufL and pufM genes were transferred into a broad-host-range vector: a derivative of pRK415 that contained a 4.2 kb EcoRI-HindIII restriction fragment and included the pufLMX genes [14]. The resulting plasmids were introduced into *C. sphaeroides* strain DD13 by conjugative crossing to produce transconjugant strains with RC-only phenotypes [11].

### 2.2. Bacterial Growth and Protein Samples Preparation

Growth of wild-type and mutant bacterial strains under dark, semiaerobic conditions was performed as previously described [14,15]. Cells were harvested and disrupted by ultrasonication; membranes for RC purification were then pelleted by ultracentrifugation. Reaction centers were solubilized from membranes with lauryldimethylamine oxide (LDAO) (Sigma-Aldrich, St. Louis, MO, USA) and purified using a polyhistidine tag (6X-His) attached to the carboxy terminus of the RC M subunit [16]. The purity of the reaction centers was estimated by absorbance spectroscopy, measuring the ratio of protein absorbance at 280 nm to bacteriochlorophyll absorbance at 802 nm (A280/A802; [17]). If the value of A280/A802 was less than 1.4, the RC sample was considered sufficiently pure for crystallization. Absorption spectra were recorded using a Shimadzu UV-1800 spectrophotometer (Shimadzu Corporation, Kyoto, Japan).

### 2.3. Studies on the Properties of Reaction Centers

Pigment extraction and pigment composition analysis of RCs were performed as previously described [18]. Thermal stability was investigated at 48 °C according to previous methodology [19], with the difference that 0.1% LDAO (Sigma-Aldrich, St. Louis, MO, USA) was used as detergent instead of 0.1% Triton X-100. The number of intact RCs in the sample was estimated by the absorption of monomeric BChl at 804 as reported [9]. The construction of the curves of absorption changes was carried out using the Origin software package (OriginLab Corporation, Northampton, MA, USA). Differential (light minus dark) absorption spectra were recorded at constant illumination with SZS-22 and KS-19 crossed light filters using a Shimadzu UV-1800 spectrophotometer (Shimadzu Corporation, Kyoto, Japan).

### 2.4. Polyacrylamide Gel Electrophoresis

Tris/MES SDS-polyacrylamide gel electrophoresis was chosen to confirm the presence of a disulfide bond between subunits in the mutant forms of RC [20], modified from [15]. It differs from conventional SDS polyacrylamide gel electrophoresis [21] by the addition of a higher percentage of polymer (acrylamide), the addition of MES (2-(N-morpholino)ethanesulfonic acid) (Sigma-Aldrich, St. Louis, MO, USA) to the buffer solution, and urea to the gels. These method alterations afford an increase in resolution. Otherwise, the protocol for setting up the experiment (polymerization of the gels, introduction of the samples, electrophoresis, staining, and washing of the gels) does not differ from the classical method. Comparatively mild denaturation conditions (30 °C, 60 min) for protein samples were used.

### 2.5. Crystallization and X-ray Diffraction Analysis

Protein crystallization was performed using vapor diffusion in a hanging drop with the addition of detergent or by *in meso* approach using a lipid sponge phase following the conditions used previously [22,23]. For the mutant forms L37Cys+L99Cys and L53Cys+L64Cys, we obtained trigonal crystals, space group P3_1_21; for the crystals of mutants L172Cys+L246Cys and M19Cys+L214Cys, space group P4_1_2_1_2; and for the crystals of the reaction center M84Cys+L278Cys, space group C2.

Samples of photosynthetic reaction centers suitable for crystallization were prepared as described [14,15]. Sample purity A280/A800 was <1.4. RC solutions with a protein concentration of 25–30 mg/mL were used.

Diffraction data for L37Cys+L99Cys, M19Cys+L214Cys, and M84Cys+L278Cys crystals were collected at the ID30A-3 beamline at the European Synchrotron Radiation Facility (ESRF), Grenoble, France [24], equipped with a Pilatus 6M detector (Dectris AG, Baden−Daettwill, Switzerland). Data collection was controlled by the MxCuBE system [25], and the strategy was calculated by BEST [26]. Data were processed and scaled using the XDS package [27].

Diffraction data for L53Cys+L64Cys and L172Cys+L246Cys crystals were collected using Proteum X8 (Bruker, Billerica, MA, USA) and XtaLAB Synergy-S (Rigaku Corporation, Tokyo, Japan) diffractometers, respectively.

Structures were solved by molecular replacement with Phaser [28], using the structure of the photosynthetic reaction center of *C. sphaeroides* strain RV [18] (PDB ID 3V3Y) as a search model. Water molecules were removed from the model. The initial model was refined using REFMAC5 [29]. Manual rebuilding of the model was performed in Coot [30]. Data statistics are summarized in Table 1 and Table 2. The figures were prepared using PyMOL [31].

## 3. Results

### 3.1. The Design and Introduction of Disulfide Bonds

Of the three protein subunits of *C. sphaeroides* RC, M and L, are pseudosymmetric with respect to each other and have 5 transmembrane α-helices, whereas subunit H has only one transmembrane α-helix, which is mostly located outside the membrane on the cytoplasmic side. This structural organization allows the introduction of disulfide bonds within one L or M subunit or between them. In the wild-type reaction center, there are several natural cysteine residues that could potentially be used for the introduction of disulfide bonds. However, because the cysteines themselves are highly reactive amino acid residues that are often important for the proper function of proteins [32], they were not used so as not to interfere with their potential role in the reaction center. In addition, it has been shown that native cysteines can be used to unidirectionally bind electrochemically active proteins to metal electrodes [33], which could be important for the development of biooptoelectronic materials and devices.

In our study, the rational design approach was employed. Using a high-resolution crystal structure of *C. sphaeroides* RC (PDB ID 6Z1J, [23]), several positions that had good geometric parameters were selected in anticipation of forming the intra- or inter-subunit S-S bonds if cysteines were placed there. When designing the mutations, the following rules were adhered to: (1) the side-chain volume of the introduced amino acids should not be significantly different from the side-chain volume of the substituted residues; (2) the side groups of the introduced cysteines should face each other to increase the probability of disulfide bond formation; and (3) in the case of proximity to electron transfer cofactors, the side groups of the substituted amino acid residues should not be turned in their direction to avoid direct influence of the cysteines on the RC function. Another important feature of the introduced substitutions was the depth of their immersion in the membrane.

**Figure 1:**
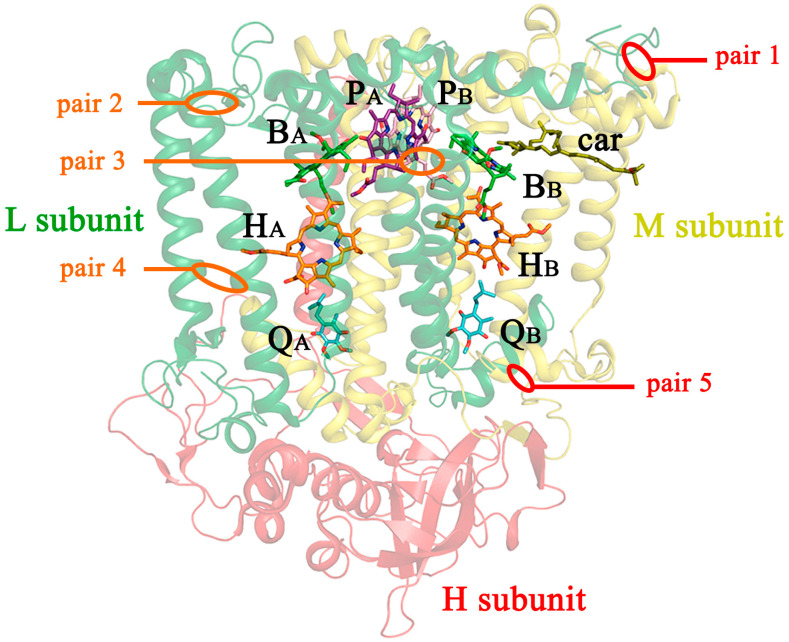
Locations of cysteine substitutions in the *C. sphaeroides* RC complex. Pairs of mutations within the subunit and between subunits are shown in orange and red, respectively. P_A_ and P_B_ are BChls of the special pair; B_A_ and B_B_ are monomeric BChl; H_A_ and H_B_ are monomeric BPheo; Q_A_ and Q_B_ are ubiquinones; car is a carotenoid. In this model, the tails of the cofactors are truncated.

Finally, the following mutation pairs were designed and obtained (Figure 1):

(1) V(M84)C+G(L278)C, periplasmic surface, intra-subunit S-S bond;
(2) A(L53)C+I(L64)C, periplasmic surface, inter-subunit S-S bond;
(3) A(L172)C+L(L246)C, membrane zone closer to periplasm, inter-subunit S-S bond;
(4) A(L37)C+S(L99)C, hydrophobic zone near BPheo H_A_, inter-subunit S-S bond;
(5) G(M19)C+T(L214)C, cytoplasmic surface, intra-subunit S-S bond.

### 3.2. Confirmation of Disulfide Bond Formation by X-ray Crystallography

**Figure 2:**
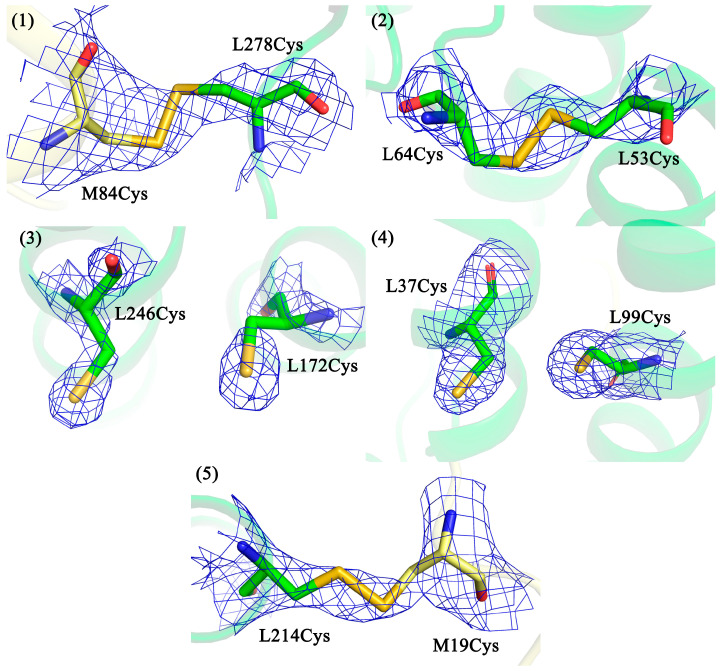
Fragments of the *2Fo-Fc* electron density maps for the crystal structures of the RC mutant forms at the sites of amino acid substitutions: (pair 1) V(M84)C+G(L278)C mutant form, 2.6 Å resolution, 0.7 σ; (pair 2) A(L53)C+I(L64)C mutant form, 2.85 Å resolution, 2.0 σ; (pair 3) A(L172)C+L(L246)C mutant form, 2.3 Å resolution, 2.1 σ; (pair 4) A(L37)C+S(L99)C mutant form, 2.6 Å resolution, 2.0 σ; (pair 5) G(M19)C+T(L214)C mutant form, 2.75 Å resolution, 1.2 σ.

In our work, X-ray crystallography was used as the main tool to observe disulfide bonds. All mutant forms were successfully crystallized, diffraction data collected, and the structures solved. Data collection and processing statistics are shown in Table 1. Apart from the sites of amino acid substitutions, no other significant changes were found in the structure of the mutant RCs compared with the wild-type. The locations of amino acid substitutions in the spatial structures of the RC mutant forms are shown in Figure 2.

Figure 2 shows that in all mutant RCs, the electron density for the introduced cysteine residues is clearly visible. It can be seen that in the cases where the mutant pairs are located closer to the protein surface, disulfide bonds are formed between the cysteines, regardless of the cytoplasmic or periplasmic side (pairs 1, 2, and 5). In the case where the mutant pairs are located deeper in the membrane part of the RC (pairs 3 and 4), no bonds are observed, and the side groups of the amino acid residues are turned away from each other.

### 3.3. Confirmation of the Formation of Disulfide Bonds by Polyacrylamide Gel Electrophoresis (PAGE)

In the inter-subunit mutants V(M84)C+G(L278)C and G(M19)C+T(L214)C (pairs 1 and 5, respectively), the electron density for the S-S bonds was visible but of poor quality and detectable only with a low cutoff. At the same time, no alternative conformations for these cysteine residues were visible either. The reasons for this could be increased mobility of the side groups of the amino acid residues at the interaction interface between the L and M subunits and/or radiation damage during the imaging process. To provide additional confirmation of the formation of these disulfide bonds, SDS-PAGE was used.

The electrophoretic method for separating the subunits of the RC has some peculiarities. The first is related to the membrane property of the protein. The bands of the RC subunits do not migrate in a gel according to their molecular weight. The characteristic letter designations of the subunits are not based on their actual molecular weight but on their electrophoretic mobility in the gel. L–light (31.4 kDa), M–medium (34.5 kDa), and H–heavy (28 kDa) subunits separate as if their molecular weights were 21, 24, and 28 kDa, respectively [17]. This is explained by the fact that the binding of charged SDS molecules to hydrophobic regions of membrane proteins is higher than the binding to amphiphilic regions of globular proteins, which affects electrophoretic mobility [34].

The second feature is that the bands of the L and M subunits disappear and the intensity of the H band increases when the RC samples are heated at 100 °C for two minutes or longer in the presence of SDS and beta-mercaptoethanol [35]. In addition, the disappearance of the bands corresponding to the L and M subunits upon heating has been described [36]. This effect was shown to be due to the aggregation of the subunits induced by beta-mercaptoethanol–they form high molecular weight aggregates that cannot penetrate the gel. It was also shown that the interactions holding the LM complex together are not disulfidic in nature.

**Figure 3:**
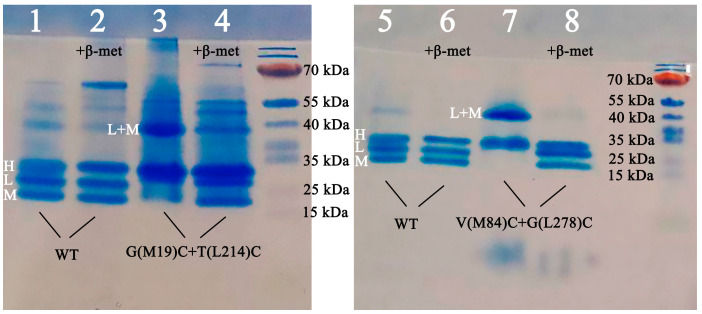
Tris/MES SDS PAGE of G(М19)С+T(L214)C and V(M84)C+G(L278)C intra-subunit disulfide mutant reaction centers. β-Mercaptoethanol (β-met) was added to the samples in lines 2, 4, 6, and 8.

Keeping in mind the abovementioned details, comparatively mild denaturation conditions were used, with a temperature of 30 °C and a denaturation time of 60 min. The results of Tris/MES SDS PAGE are shown in Figure 3.

In the wild-type RC, the formation of three bands corresponding to the RC subunits is observed (lines 1, 2, 5, and 6). In the samples containing beta-mercaptoethanol (lines 2 and 6), the band boundaries are more pronounced than without the reducing agent (lines 1 and 5).

In the G(М19)С+T(L214)C mutant RC, the formation of the LM complex is observed in the gel without beta-mercaptoethanol (line 3). Upon addition of beta-mercaptoethanol, this complex breaks down and three bands become visible, each corresponding to one of the RC subunits (line 4). 

For the mutant form V(M84)C+G(L278)C, the same picture is obtained: in the gel without beta-mercaptoethanol, the formation of the LM complex is observed (line 7), and in the presence of beta-mercaptoethanol, the complex breaks down and three bands, each corresponding to one of the RC subunits, become visible (line 8).

Summarizing the results of the electrophoresis, it can be concluded that the breakdown of the LM complex in the mutant forms (lines 4 and 8) is due to the addition of beta-mercaptoethanol being associated with the breaking of the disulfide bond that appeared as a result of the cysteine pair introduction.

### 3.4. Pigment Content and Photochemical Properties of Mutant RCs

The wild-type reaction center contains two BPheo molecules and four BChl molecules. The pigment composition remained unchanged in all investigated RC mutant forms according to the performed pigment analysis.

**Figure 4:**
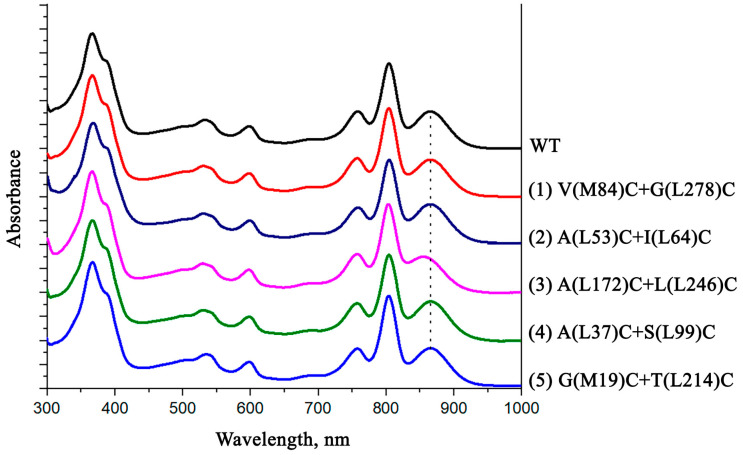
Electronic ground-state absorption spectra of the wild-type *С. sphaeroides* reaction center and its mutant forms. Spectra were measured at room temperature and normalized at the Q_Y_ H absorption band.

The absorption spectra of the isolated reaction centers are shown in Figure 4.

In the absorption spectrum of the isolated wild-type RC, shown in Figure 4, a long-wavelength band of Q_Y_ P with a maximum peak at 865 nm is accounted by the absorption of bacteriochlorophyll dimer P. The Q_Y_ В band with the maximum peak at 804 nm represents an absorption of BChl monomers and also a high-energy transition in the primary electron donor molecule. A Q_Y_ H band with a maximum at 760 nm corresponds to the absorption of bacteriopheophytin molecules. In the short wavelength region of the spectrum, the band at 599 nm reflects Q_X_ transitions in BChl molecules. At 532 nm is the Q_X_ H maximum, which corresponds to the absorption band of BPheo molecules in active and inactive electron transport chains. The shoulder at 500 nm is assigned to the carotenoid molecule. In the short-wavelength region of the absorption spectrum of isolated RCs, there is a Soret band with a maximum at 363 nm and a shoulder at the long-wavelength slope of the band at 390 nm, which reflects the absorption of all bacteriochlorins of RCs.

As can be seen from the absorption spectra shown, the introduced mutations have no significant effect on the position and amplitude of the absorption bands of the pigments. The only exception is the A(L172)C+L(L246)C mutant form, in the absorption spectrum of which a short-wavelength shift of the long-wavelength maximum of the special pair absorption band is observed. It is likely that the reason for these changes may be associated with the location of one of the introduced cysteines (L172) in close proximity to the histidine amino acid residue L173, which acts as a ligand for the magnesium atom of BChl P_A_.

**Figure 5:**
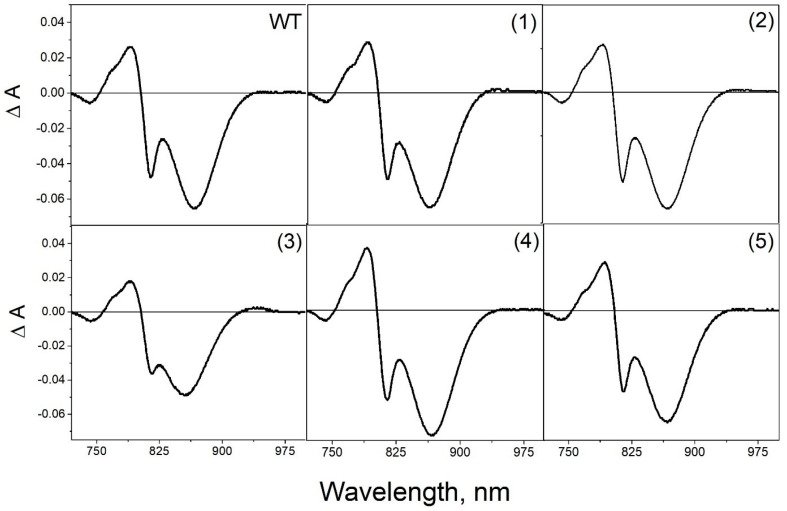
Differential (light minus dark) absorption spectra of wild-type RC *С. sphaeroides* and mutant reaction centers: (1) V(M84)C+G(L278)C, (2) A(L53)C+I(L64)C, (3) A(L172)C+L(L246)C, (4) A(L37)C+S(L99)C, and (5) G(M19)C+T(L214)C. Spectra were measured at room temperature and normalized at the Q_Y_ H absorption band.

In the differential spectra of the wild-type RC, light-induced formation of the state P^+^Q_A_^–^ causes bleaching of the Q_Y_ P band at 865 nm, a short-wavelength shift of the Q_Y_B band, and a long-wavelength shift of the Q_Y_ H band (Figure 5). Similar spectral changes were observed upon illumination of all mutant reaction centers, demonstrating effective electron transfer from the primary donor P to the acceptor Q_A_. 

As can be seen from the differential (light minus dark) absorption spectra, the introduced mutations have no significant effect on the photochemical properties of mutant RCs. Similar to what was observed in the absorption spectra, the amplitude of the Q_Y_ P band was noticeably reduced in the RC A(L172)C+L(L246)C. As mentioned above the possible reason for these changes may be associated with the location of one of the introduced cysteines (L172) in close proximity to a ligand for the magnesium atom of BChl P_A_. 

### 3.5. Thermostability of the Mutant RCs

**Figure 6:**
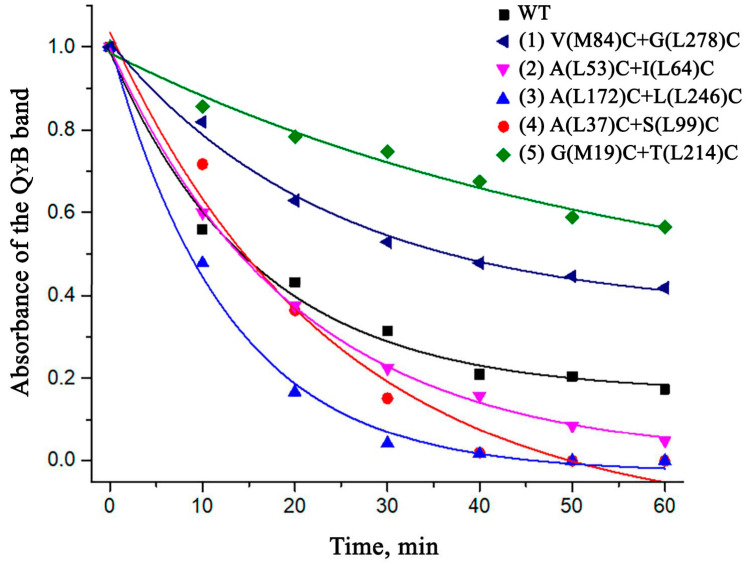
Thermodependent changes in the long-wavelength absorption band of monomeric BChls (at an incubation temperature of 48 °C).

Since the main goal of this research was to stabilize the RCs, the thermal stability of the isolated mutant pigment-protein complexes was investigated (Figure 6).

According to the data obtained, the mutations made for the formation of intra-subunit disulfide bridges (A(L37)C+S(L99)C, A(L172)C+L(L246)C, and A(L53)C+I(L64)C) did not contribute positively to the stabilization of the RC but, on the contrary, had a destabilizing effect on the structure of the complex. However, it was observed that the mutations between the L and M subunits (G(М19)С+T(L214)C and V(M84)C+G(L278)C) significantly contributed to the stabilization of the RC. After 60 min of incubation, more than 50% of the native complexes remained in the sample in the case of G(М19)С+T(L214)C and more than 40% in the case of V(M84)C+G(L278)C, which was significantly more compared to about 20% of the undenatured wild-type RCs under the same conditions.

## 4. Discussion

The introduction of disulfide bonds into protein molecules was previously reported within the literature as a method to increase their overall structural stability [37,38,39]. A disulfide bond formed between the thiol groups of two spatially close cysteine residues is often important for protein folding, stability, and function [40,41]. Due to conformational entropy, native disulfide bonds stabilize the conformation of protein molecules [42], whereas removal of native disulfides can result in decreased stability of the target protein [43]. Previous studies have shown that the proper introduction of disulfide bonds can stabilize the flexible region of target proteins and reduce conformational entropy by fixing the protein in a single desired conformation [44].

This approach is more commonly used with globular proteins but is also possible with integral membrane proteins. The major developments of this method are in the area of stabilization of G protein-coupled receptors (GCPRs), also known as seven-(pass)-transmembrane domain receptors. For example, a double cysteine mutant of the opsin form of rhodopsin was obtained in which the formation of a disulfide bond between the introduced amino acid residues was observed [45], which was later confirmed by the crystal structure [46]. The formation of the S-S bond resulted in an increase in thermal stability and had a minor effect on the functional properties of the protein. Another example of a GCPR whose thermal stability was improved by the introduction of a disulfide bond is the serotonin 5-HT_2C_ receptor [47].

To our knowledge, the possibility of stabilization of the photosynthetic reaction center by disulfide bridges has not been previously studied. The reaction centers, unlike GCPRs, are complexes with multiple subunits. Therefore, it was chosen to introduce mutations both within one subunit and between two subunits.

In two intra-subunit mutant forms A(L172)C+L(L246)C and A(L37)C+S(L99)C (pairs 3 and 4, respectively), the formation of disulfide bonds did not occur. This can be attributed to the fact that these cysteine pairs were too deeply immersed in the membrane and were therefore inaccessible to the bacterial disulfide bond formation systems [48] that promote the oxidation process. Considering these data and the fact that in rhodopsin, successful S-S bond formation also occurred near the surface [46], it was confirmed that proximity to the extramembranous part of the integral membrane protein is essential for S-S bond formation.

In three mutant forms V(M84)C+G(L278)C, A(L53)C+I(L64)C and G(M19)C+T(L214)C (pairs 1, 2, and 5, respectively), in which the introduced cysteines were close to the protein surface, disulfide bonds were formed regardless of whether these amino acid residues were on the cytoplasmic side or on the periplasmic surface. It should be noted that no specific oxidizing agents were used during bacterial growth or protein purification to stimulate S-S bond formation.

All inter-subunit mutant forms were less thermally stable than wild-type RCs. However, the cysteine pair L53Cys-L64Cys (pair 2), which formed a disulfide bond, had the least destabilizing effect on the structure of the complex compared with cysteine pairs that did not form S-S bridges.

In the case of mutant RCs with inter-subunit S-S bonds V(M84)C+G(L278)C and G(M19)C+T(L214)C (pairs 1 and 5, respectively), both complexes exhibited increased thermal stability compared with wild-type RCs. It is assumed that such strengthening of the RC structure occurs due to the strengthening of the complex at the level of the quaternary structure of the protein.

The genetically modified reaction centers described in this work retained the ability for photoinduced electron transfer from the primary electron donor molecule (P) to the electron acceptor molecules (Q). This implies that the mutant RCs V(M84)C+G(L278)C and G(M19)C+T(L214)C with enhanced thermal stability could potentially be used in photo-bioelectrochemical systems where wild-type reaction centers are used. Examples of which include the sunlight-driven online sensing of various toxic compounds [49] and bio-hybrid systems that have been shown to be effective transducers of solar radiation [50,51]. Bio-hybrids can also be used as materials for biooptoelectronics [52,53], functionally integrated into devices [54], and used as active elements in bio-photonic energy cells [6]. Due to their increased structural stability, the V(M84)C+G(L278)C and G(M19)C+T(L214)C mutants could be more effective for the processes mentioned above, which is only a speculation for now and requires further research and confirmation.

In summary, the data obtained in this work demonstrate the possibility of introducing disulfide bonds into bacterial photosynthetic reaction centers to increase their thermal stability without loss of functional activity. The reaction center is a representative of integral membrane proteins with an α-helical structure. This is the most common type of transmembrane protein. In humans, for example, an estimated 27% of all proteins are α-helical membrane proteins [55]. This class includes many other IMPs of interest to the scientific community, where the introduction of disulfide bonds may be an effective stabilization mechanism. We believe that our results may be useful for the future development of multisubunit α-helical integral membrane protein complexes with enhanced stability.

## Figures and Tables

**Table 1:** Data collection and processing.

	*L37Cys+L99Cys*	*L53Cys+L64Cys*	*L172Cys+L246Cys*	*M19Cys+L214Cys*	*M84Cys+L278Cys*
Diffraction source	ESRF, beamline ID30A-3	Proteum X8 (Bruker)	XtaLAB Synergy-S (Rigaku)	ESRF, beamline ID30A-3	ESRF, beamline ID30A-3
Wavelength (Å)	0.9677	1.54178	1.54178	0.9677	0.9677
Temperature (K)	100	110	120	100	100
Detector	DECTRIS Eiger X 4M	PLATINUM135 CCD	HyPix-6000C	DECTRIS Eiger X 4M	DECTRIS Eiger X 4M
Rotation range per image (°)	0.15	0.5	0.5	0.05	0.1
Total rotation range (°)	150	180	120	50	200
Space group	P3_1_21	P3_1_21	P4_1_2_1_2	P4_1_2_1_2	C2
*a*, *b*, *c* (Å), α, β, γ, ◦	139.8 139.8 186.5 90 90 120	139.6 139.6 185.0 90 90 120	99.7 99.7 239.1 90 90 90	100.91 100.91 237.0 90 90 90	253.1 75.9 65.8 90.0 95.5 90.0
Resolution range (Å)	30.00–2.60 (2.67–2.60)	30.00–2.85 (2.95–2.85)	30.00–2.30 (2.40–2.30)	30.00–2.75 (2.82–2.75)	30.00–2.60 (2.67–2.60)
Total No. of reflections	564 510 (43 474)	283 148 (16 729)	366 770 (30 085)	115 943 (8 973)	150 052 (11 638)
No. of unique reflections	65 303 (4 784)	49 267 (4 765)	53 136 (5 315)	32 119 (2 369)	38 037 (2 832)
Completeness (%)	99.9 (100.0)	99.7 (97.6)	97.3 (82.7)	98.1 (99.9)	98.7 (99.3)
Redundancy	8.6 (9.1)	5.7 (3.5)	6.9 (5.7)	3.6 (3.8)	3.9 (4.1)
〈*I*/σ(*I*)〉	10.13 (1.42)	5.42 (1.79)	8.26 (1.10)	7.92 (1.06)	8.15 (1.02)
*R*_r.i.m._‡	17.3 (161.4)	27.1 (58.2)	11.7 (65.8)	11.9 (124.4)	9.8 (134.9)
CC_1/2_	99.7 (57.9)	99.6 (59.4)	96.6 (60.2)	99.6 (40.1)	99.7 (58.6)

**Table 2:** Structure solution and refinement.

	*L37Cys+L99Cys*	*L53Cys+L64Cys*	*L172Cys+L246Cys*	*M19Cys+L214Cys*	*M84Cys+L278Cys*
Resolution range (Å)	30.00–2.60 (2.64–2.60)	30.00–2.85 (2.95–2.85)	30.00–2.30 (2.40–2.30)	46.0–2.75 (2.82–2.75)	41.00–2.60 (2.67–2.60)
Completeness (%)	99.9 (100.0)	99.5 (96.1)	99.3 (99.0)	98.0 (96.0)	98.6 (99.0)
No. of reflections, working set	65 301 (2 567)	48 500 (2 565)	44 919 (2 614)	32 091 (3 403)	38 001 (3 296)
No. of reflections, test set	3 351 (153)	2 435 (131)	2 273 (140)	1 282 (141)	1 519 (138)
*R*_cryst_	18.56 (25.95)	22.85 (33.77)	25.86 (26.08)	19.91 (34.63)	19.27 (40.40)
*R*_free_	20.74 (30.34)	27.90 (38.49)	30.82 (32.56)	27.06 (42.24)	24.93 (40.01)
R.m.s. deviations					
Bonds (Å)	0.009	0.010	0.010	0.010	0.009
Angles (°)	1.122	1.314	1.354	1.234	1.159
PDB ID	8C5X	8C6K	8C87	8C88	8C7C

## Data Availability

MDPI Research Data Policies.

## References

[1] Allen J.P., Feher G., Yeates T.O., Komiya H., Rees D.C. (1987). Structure of the Reaction Center from Rhodobacter Sphaeroides R-26: The Cofactors. Proc. Natl. Acad. Sci. USA.

[2] Kálmán L., Williams J.C., Allen J.P. (2008). Comparison of Bacterial Reaction Centers and Photosystem II. Photosynth. Res..

[3] Pakula A.A., Sauer R.T. (1989). Genetic analysis of protein stability and function. Annu. Rev. Genet..

[4] McPherson A., Gavira J.A. (2014). Introduction to Protein Crystallization. Acta Cryst. F.

[5] Wraight C.A., Clayton R.K. (1974). The Absolute Quantum Efficiency of Bacteriochlorophyll Photooxidation in Reaction Centres of Rhodopseudomonas Spheroides. Biochim. Biophys. Acta BBA–Bioenerg..

[6] Grattieri M., Beaver K., Gaffney E.M., Dong F., Minteer S.D. (2020). Advancing the Fundamental Understanding and Practical Applications of Photo-Bioelectrocatalysis. Chem. Commun..

[7] Fufina T.Y., Vasilieva L.G. (2021). Effect of Detergents and Osmolytes on Thermal Stability of Native and Mutant Rhodobacter Sphaeroides Reaction Centers. Biochem. Mosc..

[8] Selikhanov G., Fufina T., Guenther S., Meents A., Gabdulkhakov A., Vasilieva L. (2022). X-ray Structure of the *Rhodobacter Sphaeroides* Reaction Center with an M197 Phe→His Substitution Clarifies the Properties of the Mutant Complex. IUCrJ.

[9] Holden-Dye K., Crouch L.I., Williams C.M., Bone R.A., Cheng J., Böhles F., Heathcote P., Jones M.R. (2011). Opposing Structural Changes in Two Symmetrical Polypeptides Bring about Opposing Changes to the Thermal Stability of a Complex Integral Membrane Protein. Arch. Biochem. Biophys..

[10] Kangur L., Jones M.R., Freiberg A. (2017). Hydrogen Bonds in the Vicinity of the Special Pair of the Bacterial Reaction Center Probed by Hydrostatic High-Pressure Absorption Spectroscopy. Biophys. Chem..

[11] Jones M.R., Visschers R.W., Van Grondelle R., Hunter C.N. (1992). Construction and Characterization of a Mutant of Rhodobacter Sphaeroides with the Reaction Center as the Sole Pigment-Protein Complex. Biochemistry.

[12] Vasilieva L.G., Bolgarina T.I., Khatynov R.A., Shkuropatov A.Y., Miyake J., Shuvalov V.A. (2001). Substitution of Valine-157 Residue by Tyrosine in the L-Subunit of the Rhodobacter Sphaeroides Reaction Center. Dokl. Biochem. Biophys..

[13] Liu H., Naismith J.H. (2008). An Efficient One-Step Site-Directed Deletion, Insertion, Single and Multiple-Site Plasmid Mutagenesis Protocol. BMC Biotechnol..

[14] Khatypov R.A., Vasilieva L.G., Fufina T.Y., Bolgarina T.I., Shuvalov V.A. (2005). Substitution of Isoleucine L177 by Histidine Affects the Pigment Composition and Properties of the Reaction Center of the Purple Bacterium Rhodobacter Sphaeroides. Biochemistry.

[15] Fufina T.Y., Vasilieva L.G., Khatypov R.A., Shkuropatov A.Y., Shuvalov V.A. (2007). Substitution of Isoleucine L177 by Histidine in *Rhodobacter Sphaeroides* Reaction Center Results in the Covalent Binding of P_A_ Bacteriochlorophyll to the L Subunit. FEBS Lett..

[16] Goldsmith J.O., Boxer S.G. (1996). Rapid Isolation of Bacterial Photosynthetic Reaction Centers with an Engineered Poly-Histidine Tag. Biochim. Biophys. Acta BBA–Bioenerg..

[17] Okamura M.Y., Steiner L.A., Feher G. (1974). Characterization of Reaction Centers from Photosynthetic Bacteria. I. Subunit Structure of the Protein Mediating the Primary Photochemistry in Rhodopseudomonas Spheroides R-26. Biochemistry.

[18] Vasilieva L.G., Fufina T.Y., Gabdulkhakov A.G., Leonova M.M., Khatypov R.A., Shuvalov V.A. (2012). The Site-Directed Mutation I(L177)H in Rhodobacter Sphaeroides Reaction Center Affects Coordination of PA and BB Bacteriochlorophylls. Biochim. Biophys. Acta BBA–Bioenerg..

[19] Fufina T.Y., Vasilieva L.G., Shuvalov V.A. (2010). Examination of Stability of Mutant Photosynthetic Reaction Center of Rhodobacter Sphaeroides I(L177)H and Determination of Location of Bacteriochlorophyll Covalently Bound to the Protein. Biochem. Mosc..

[20] Kashino Y., Koike H., Satoh K. (2001). An Improved Sodium Dodecyl Sulfate-Polyacrylamide Gel Electrophoresis System for the Analysis of Membrane Protein Complexes. Electrophoresis.

[21] Laemmli U.K. (1970). Cleavage of Structural Proteins during the Assembly of the Head of Bacteriophage T4. Nature.

[22] Gabdulkhakov A.G., Fufina T.Y., Vasilieva L.G., Mueller U., Shuvalov V.A. (2013). Expression, Purification, Crystallization and Preliminary X-Ray Structure Analysis of Wild-Type and L(M196)H-Mutant *Rhodobacter Sphaeroides* Reaction Centres. Acta Crystallogr. Sect. F Struct. Biol. Cryst. Commun..

[23] Selikhanov G., Fufina T., Vasilieva L., Betzel C., Gabdulkhakov A. (2020). Novel Approaches for the Lipid Sponge Phase Crystallization of the *Rhodobacter Sphaeroides* Photosynthetic Reaction Center. IUCrJ.

[24] Nurizzo D., Mairs T., Guijarro M., Rey V., Meyer J., Fajardo P., Chavanne J., Biasci J.-C., McSweeney S., Mitchell E. (2006). The ID23-1 Structural Biology Beamline at the ESRF. J. Synchrotron Radiat..

[25] Gabadinho J., Beteva A., Guijarro M., Rey-Bakaikoa V., Spruce D., Bowler M.W., Brockhauser S., Flot D., Gordon E.J., Hall D.R. (2010). *MxCuBE*: A Synchrotron Beamline Control Environment Customized for Macromolecular Crystallography Experiments. J. Synchrotron Radiat..

[26] Bourenkov G.P., Popov A.N. (2010). Optimization of Data Collection Taking Radiation Damage into Account. Acta Crystallogr. Sect. D Biol. Crystallogr..

[27] Kabsch W. (2010). *XDS*. Acta Crystallogr. Sect. D Biol. Crystallogr..

[28] McCoy A.J., Grosse-Kunstleve R.W., Adams P.D., Winn M.D., Storoni L.C., Read R.J. (2007). *Phaser* Crystallographic Software. J. Appl. Crystallogr..

[29] Murshudov G.N., Skubák P., Lebedev A.A., Pannu N.S., Steiner R.A., Nicholls R.A., Winn M.D., Long F., Vagin A.A. (2011). *REFMAC* 5 for the Refinement of Macromolecular Crystal Structures. Acta Crystallogr. Sect. D Biol. Crystallogr..

[30] Emsley P., Lohkamp B., Scott W.G., Cowtan K. (2010). Features and Development of *Coot*. Acta Crystallogr. Sect. D Biol. Crystallogr..

[31] Delano W.L. (2022). The PyMOL Molecular Graphics System. http://www.pymol.org.

[32] Netto L.E.S., de Oliveira M.A., Monteiro G., Demasi A.P.D., Cussiol J.R.R., Discola K.F., Demasi M., Silva G.M., Alves S.V., Faria V.G. (2007). Reactive Cysteine in Proteins: Protein Folding, Antioxidant Defense, Redox Signaling and More. Comp. Biochem. Physiol. Part C Toxicol. Pharmacol..

[33] Reiss B.D., Hanson D.K., Firestone M.A. (2007). Evaluation of the Photosynthetic Reaction Center Protein for Potential Use as a Bioelectronic Circuit Element. Biotechnol. Prog..

[34] Rath A., Glibowicka M., Nadeau V.G., Chen G., Deber C.M. (2009). Detergent Binding Explains Anomalous SDS-PAGE Migration of Membrane Proteins. Proc. Natl. Acad. Sci. USA.

[35] Clayton R.K., Haselkorn R. (1972). Protein Components of Bacterial Photosynthetic Membranes. J. Mol. Biol..

[36] Shepherd W.D., Kaplan S. (1978). Effect of Heat and 2-Mercaptoethanol on Intracytoplasmic Membrane Polypeptides of Rhodopseudomonas Sphaeroides. J. Bacteriol..

[37] Liu H.-X., Li L., Yang X.-Z., Wei C.-W., Cheng H.-M., Gao S.-Q., Wen G.-B., Lin Y.-W. (2019). Enhancement of Protein Stability by an Additional Disulfide Bond Designed in Human Neuroglobin. RSC Adv..

[38] van Beek H.L., Wijma H.J., Fromont L., Janssen D.B., Fraaije M.W. (2014). Stabilization of Cyclohexanone Monooxygenase by a Computationally Designed Disulfide Bond Spanning Only One Residue. FEBS Open Biol..

[39] Sanchez-Romero I., Ariza A., Wilson K.S., Skjøt M., Vind J., De Maria L., Skov L.K., Sanchez-Ruiz J.M. (2013). Mechanism of Protein Kinetic Stabilization by Engineered Disulfide Crosslinks. PLoS ONE.

[40] Creighton T.E., Zapun A., Darby N.J. (1995). Mechanisms and Catalysts of Disulphide Bond Formation in Proteins. Trends Biotechnol..

[41] Fass D. (2012). Disulfide Bonding in Protein Biophysics. Annu. Rev. Biophys..

[42] Dill K.A. (1990). Dominant Forces in Protein Folding. Biochemistry.

[43] Liu D., Cowburn D. (2016). Combining Biophysical Methods to Analyze the Disulfide Bond in SH2 Domain of C-Terminal Src Kinase. Biophys. Rep..

[44] Matsumura M., Signor G., Matthews B.W. (1989). Substantial Increase of Protein Stability by Multiple Disulphide Bonds. Nature.

[45] Xie G., Gross A.K., Oprian D.D. (2003). An Opsin Mutant with Increased Thermal Stability. Biochemistry.

[46] Standfuss J., Xie G., Edwards P.C., Burghammer M., Oprian D.D., Schertler G.F.X. (2007). Crystal Structure of a Thermally Stable Rhodopsin Mutant. J. Mol. Biol..

[47] Popov P., Peng Y., Shen L., Stevens R.C., Cherezov V., Liu Z.-J., Katritch V. (2018). Computational Design of Thermostabilizing Point Mutations for G Protein-Coupled Receptors. eLife.

[48] Landeta C., Boyd D., Beckwith J. (2018). Disulfide Bond Formation in Prokaryotes. Nat. Microbiol..

[49] Grattieri M., Minteer S.D. (2018). Self-Powered Biosensors. ACS Sens..

[50] Kornienko N., Zhang J.Z., Sakimoto K.K., Yang P., Reisner E. (2018). Interfacing Nature’s Catalytic Machinery with Synthetic Materials for Semi-Artificial Photosynthesis. Nat. Nanotech..

[51] Grattieri M. (2020). Purple Bacteria Photo-Bioelectrochemistry: Enthralling Challenges and Opportunities. Photochem. Photobiol. Sci..

[52] Hasan K., Grippo V., Sperling E., Packer M.A., Leech D., Gorton L. (2017). Evaluation of Photocurrent Generation from Different Photosynthetic Organisms. ChemElectroChem.

[53] Milano F., Punzi A., Ragni R., Trotta M., Farinola G.M. (2019). Photonics and Optoelectronics with Bacteria: Making Materials from Photosynthetic Microorganisms. Adv. Funct. Mater..

[54] Kruse O., Rupprecht J., Mussgnug J.H., Dismukes G.C., Hankamer B. (2005). Photosynthesis: A Blueprint for Solar Energy Capture and Biohydrogen Production Technologies. Photochem. Photobiol. Sci..

[55] Almén M.S., Nordström K.J., Fredriksson R., Schiöth H.B. (2009). Mapping the Human Membrane Proteome: A Majority of the Human Membrane Proteins Can Be Classified According to Function and Evolutionary Origin. BMC Biol..

